# Long-range structural and magnetic coherence in embedded mesospin metamaterials

**DOI:** 10.1038/s41598-026-48207-w

**Published:** 2026-04-12

**Authors:** Christina Vantaraki, Oier Bikondoa, Matías P. Grassi, Brindaban Ojha, Alkaios Stamatelatos, Natalia Kwiatek-Maroszek, Miguel Angel Niño Orti, Michael Foerster, Thomas Saerbeck, Daniel Primetzhofer, Max Wolff, Nicolas Jaouen, Thomas P. A. Hase, Vassilios Kapaklis

**Affiliations:** 1 Department of Physics and Astronomy, Uppsala University, Box 516, 75120 Uppsala, Sweden; 2 Department of Physics, University of Warwick, Coventry, CV4 7AL UK; 3 ALBA Synchrotron Light Facility, Cerdanyola del Valles, 08290 Barcelona, Spain; 4 Instituto de Química Física “Blas Cabrera”, CSIC, 28006 Madrid, Spain; 5 Institut Laue-Langevin, Grenoble, France; 6 Synchrotron SOLEIL, L’Orme des Merisiers Saint-Aubin, 91192 Gif-sur-Yvette, France

**Keywords:** Materials science, Physics

## Abstract

Engineered assemblies of interacting magnetic elements—magnetic metamaterials—provide a powerful route to tailor collective magnetic order and dynamics. By structuring matter at the mesoscale, they bridge atomic magnetism and macroscopic functionality, enabling emergent behaviour inaccessible in conventional materials. However, realizing large-area metamaterials that combine high morphological uniformity with intrinsic long-range order has remained challenging, largely due to the structural disorder inherent to lithographic fabrication. Here we demonstrate a scalable route to structurally and magnetically coherent metamaterials by embedding iron-ions to form mesospins within a non-magnetic thin film palladium host matrix. Using controlled implantation, we realize morphologically uniform arrays that spontaneously develop extended antiferromagnetic order in the as-fabricated state—without the need of external annealing or field cycling. Resonant X-ray scattering and microscopy reveal sharp magnetic Bragg peaks modulated by the mesospin form factor, evidencing long-range antiferromagnetic order coupled to structural coherence. This embedded architecture establishes a platform for exploring coherent spin–photon interactions and functional X-ray scattering in magnetic metamaterials free from lithographic topography and disorder.

## Introduction

Magnetic metamaterials have emerged as a versatile platform for exploring how geometry, dimensionality, and designed interactions govern collective magnetic behavior^1–5^. Through tailored arrangements of interacting mesoscopic elements, systems can be designed to incorporate frustrated^6^ and correlated magnetic states^3^ that bridge atomic-scale magnetism and macroscopic functionality. This capacity to engineer interactions at the mesoscale has enabled advances in thermally driven ordering^7–9^, magnetic phases and kinetics defined by topology^10,11^, reconfigurable spin textures^12,13^, magnonic circuitry^14,15^, reconfigurable logic^16–18^ and hybrid magneto-optical systems^19,20^, where geometry and coupling strength can be tuned independently of intrinsic material parameters. Despite this progress, fabricating large-area arrays that combine morphological uniformity with intrinsic long-range magnetic order remains challenging. Conventional lithographic routes introduce edge roughness, inter-element variability, and chemical inhomogeneity that disrupt long-range correlations and obscure intrinsic magnetic order. These imperfections often require post-growth annealing or field cycling to reveal ordered states, potentially masking the spontaneous emergence of collective order. Conventional lithographically defined systems are also constrained by the intrinsic properties of the base materials from which the metamaterials are fabricated. Key parameters–such as the magnetic ordering temperature, anisotropy, or moment magnitude–can typically only be modified indirectly, for instance through finite-size effects or careful control during film deposition. As a result, the available design space is constrained, and the functional versatility of the resulting metamaterials limited.

**Fig. 1:**
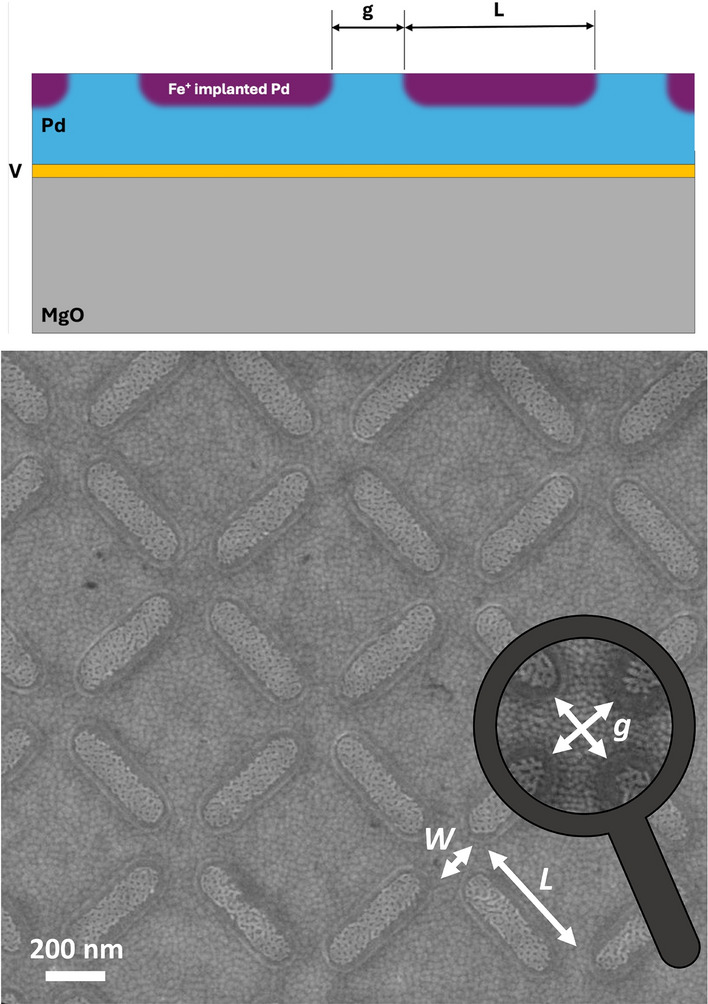
Implanted magnetic metamaterial. Top: schematic cross-section of the implanted metamaterial architecture^25^. Bottom: scanning electron microscopy image of a square artificial spin ice patterned via ion implantation, illustrating the high uniformity of ion-implanted mesospins. The surface morphology of the array reflects the patterned implantation mask, and a slight surface swelling of the implanted regions is visible due to the local lattice expansion induced by Fe incorporation. The mesospins of Fe-implanted regions in a paramagnetic Pd matrix, have lateral dimensions of $L=$470 nm, $W=$170 nm and an edge-to-edge gap of $g=$170 nm. Details of the sample fabrication are presented in “Methods”.

Advances in resonant and coherent X-ray scattering now offer a powerful route to probe magnetic metamaterials with high sensitivity to both chemical and magnetic correlations. These techniques enable direct access to spin textures, interference effects, and topological configurations that remain otherwise hidden in standard dichroic approaches^19,20^. Fully exploiting this capability, however, requires metamaterials with well-defined morphology and robust long-range magnetic order–conditions that enhance coherence, sharpen reciprocal-space features, and expose the underlying structure–factor physics. This has intensified the need for material architectures that are intrinsically compatible with coherent scattering approaches and capable of supporting reproducible emergent magnetic order across extended areas.

Here, we present a scalable approach to fabricating coherent magnetic metamaterials by embedding ferromagnetic mesospins within a non-magnetic host matrix. For demonstration, we employ a square artificial spin ice (ASI) geometry—a popular lattice with a well-defined antiferromagnetic ground state (Fig. 1)^3,5,9^. Controlled Fe$^+$ ion implantation into Pd thin films produces single-domain mesospins that spontaneously assemble into extended antiferromagnetic domains in the *as-implanted* state. Relative to our earlier implantation studies that established fabrication and real-space magnetic textures^25,26^, the present work provides a reciprocal-space demonstration of simultaneous structural and magnetic long-range coherence. Resonant X-ray reflectometry, polarized neutron reflectometry, and photoemission electron microscopy establish the structural and magnetic depth profiles, while resonant soft X-ray diffraction reveals sharp structural Bragg peaks together with mixed-parity magnetic reflections and a clearly resolved mesospin-basis (“$\times$”) envelope. Together, these findings establish a class of structurally embedded magnetic metamaterials with intrinsic long-range order, providing a basis for quantitatively interpretable, geometry- and form-factor-encoded scattering studies of collective magnetism and coherent photon–spin coupling.

##  Results and discussion

###  Structural and magnetization depth profiles

**Fig. 2:**
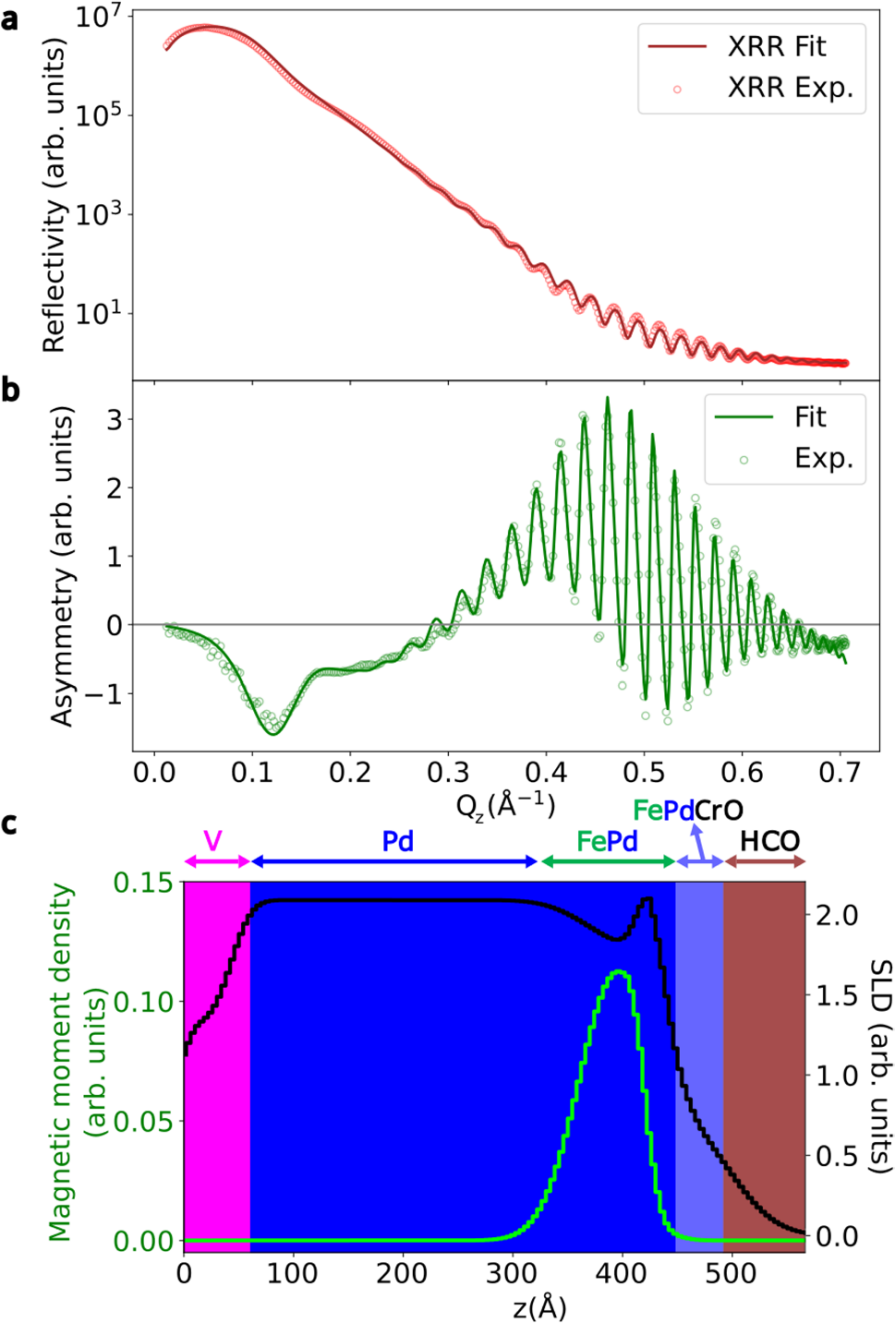
Resonant X-ray reflectivity and magnetic moment profiles. (**a**) X-ray reflectivity curve. (**b**) Asymmetry obtained from the subtraction of XRR signals acquired with opposite in-plane magnetic fields with a beam energy of 707 eV, sensitive to Fe. (**c**) Electron density (black line) and magnetic moment density (green line) extracted from simultaneously fitting the reflectivity and asymmetry data for different X-ray photon energies. The depth profiles originate at the MgO substrate ($z = 0$), followed by the V adhesion layer, Pd film, and Fe-implanted region. These layer positions are indicated in the panel for clarity.

To establish the structural and magnetization depth profiles of the implanted films, we employed resonant X-ray reflectivity (XRR) and polarized neutron reflectivity (PNR) on continuous-film samples. Palladium lies close to the Stoner criterion for ferromagnetism and can become magnetically polarized when surrounded by magnetic impurities^21–23^. Figure 2a, b show the total reflectivity and asymmetry signals for left ($I^{L}$) and right ($I^{R}$) circularly polarized X-rays, measured at the Fe $L_{3}$ edge (707 eV) from the sample with a nominal 40 nm Pd film. A simultaneous fit of these data to a single model yields both the electron density (scattering-length density, SLD) and the Fe magnetic moment density profiles, presented in Fig. 2c. These profiles reproduce the expected layer sequence: MgO substrate, V adhesion layer, Pd film, Fe-implanted region, a thin PdCrO layer, and a surface water/hydrocarbon contamination layer–demonstrating that the implantation process preserves the overall film morphology. The apparent thickness of the contamination layer in Fig. 2c may be somewhat overestimated, as the fitting model can also capture contributions from top-layer roughness induced by ion bombardment during the ion implantation process (see Fig. 1). The Fe concentration peaks near the surface and extends approximately 15 nm into the Pd layer. The magnetic moment profile shows a slightly asymmetric but well-defined distribution, peaking roughly 5 nm below the surface with a width of about 7 nm. A notable feature is the dip in SLD, and therefore in electron density, within the mixed Pd:Fe region (Fig. 2c). In resonant reflectivity this effective SLD is energy dependent and includes dispersive corrections ($f'$, $f''$) near the Fe $L_{3}$ edge; accordingly, the dip reflects both the implantation-driven composition/density change and the resonant modification of the complex atomic scattering factors at the measurement energy. This subtle difference in electron density between the Pd matrix and the Fe-implanted regions provides the contrast used to explore the implanted metamaterials.

Polarised neutron reflectometry analyses (Supplementary Information) reproduce similar magnetic profiles, but in this case the magnetic region not only coincides with the implanted Fe distribution but also includes contributions from induced Pd polarization, consistent with previous ion-beam studies^24–26^. The two probes are therefore complementary: resonant X-ray methods at the Fe edge are Fe-selective, whereas PNR reports the total magnetic depth profile (Fe+Pd). For these implantation-defined structures, resonant XRR/PNR comparison is most physically meaningful at the level of the extracted chemical and magnetic SLD depth profiles, rather than by tabulating single thickness/roughness values that act only as model-dependent effective parameters for graded interfaces. A key finding, when considering both the X-ray and neutron data (Supplementary Information), is the consistency between two independently prepared samples, probed using distinctly different techniques, which demonstrates the reproducibility of the ion implantation process in generating well-defined magnetic volumes close to the surface.

###  Magnetic microscopy

**Fig. 3:**
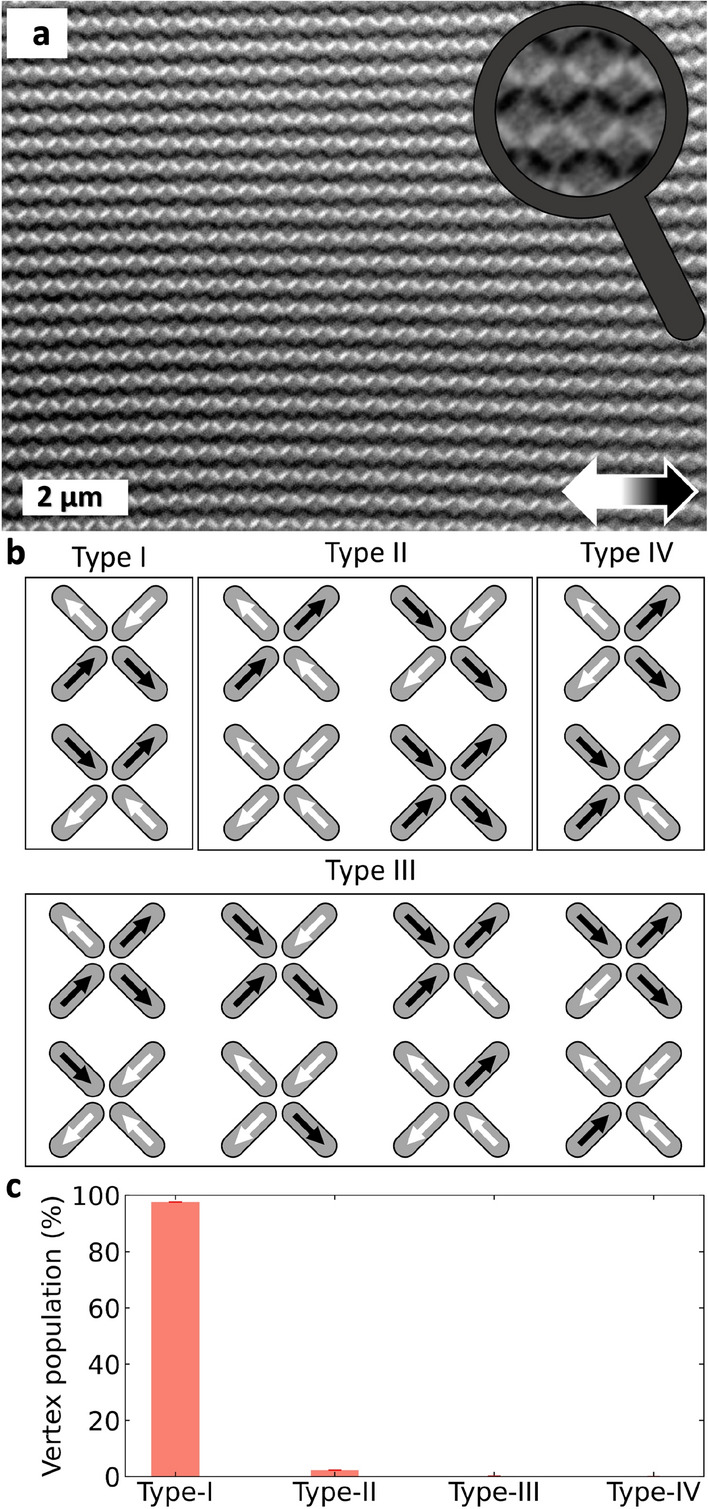
Real-space magnetic imaging and vertex populations. (**a**) Representative PEEM - XMCD image of the implanted square ASI lattice in the as-implanted state. The black and white colors indicate a magnetization component parallel and antiparallel to the X-ray beam, respectively. (**b**) Vertex types for the square ASI lattice. Type-I has the lowest energy state, followed by Type-II, III and IV. (**c**) Vertex population for the implanted square ASI lattice. A dominant Type-I configuration of the ASI lattice is observed, consistent with the expected nearest-neighbour square-ASI ground state and with the direct PEEM–XMCD real-space evidence of extended ordered domains, enabling the use of these samples as benchmarks in X-ray scattering studies.

**Fig. 4:**
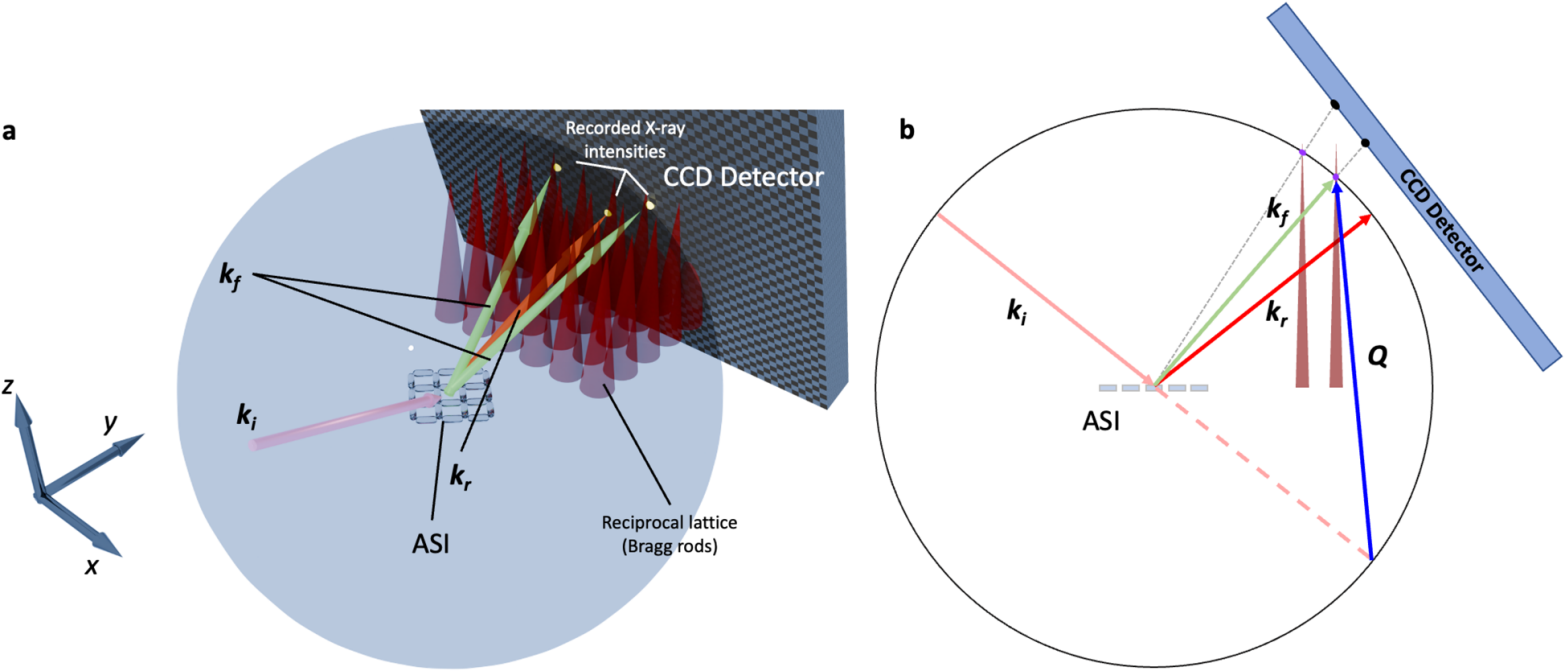
X-ray scattering from implanted ASIs. (**a**) Ewald construction illustrating the scattering geometry in the soft X-ray diffraction experiments on the implanted artificial spin ice lattices. The reciprocal lattice of the ASI gives rise to a series of intensity rods (schematically depicted as cones) extending along the out-of-plane direction. The intensity modulation along these rods encodes information about the vertical structure of the mesospins. The incident beam vector ${\bf k}_i$, reflected beam ${\bf k}_r$, and scattered beam ${\bf k}_f$ define the scattering geometry, with the end of ${\bf k}_f$ lying on the surface of the Ewald sphere. A constructive scattering condition is satisfied whenever the Ewald sphere intersects an intensity rod. The recorded diffraction intensities are determined not only by the structural contribution but also by the detector position (gray box), the form factor of the implanted mesospins, and the specific magnetization textures within the elements. (**b**) Side view (*yz* plane) of the Ewald construction, showing the intersection of the Ewald sphere with the Bragg rods and the resulting mapping of diffraction peaks onto the detector plane.

Having established the magnetic depth profile of the continuous films, we examine the in-plane magnetic ordering in the implanted array. Photoemission electron microscopy with X-ray magnetic circular dichroism (PEEM–XMCD) provides direct, element-specific imaging of the magnetization at the mesoscale (Fig. 3a). The PEEM image shows that each implanted element exhibits uniform contrast, confirming single-domain behavior. Moreover, the elements form extended antiferromagnetic domains corresponding to the Type-I ground state of the square geometry (Fig. 3b). The observation of large, defect-free domains indicates strong inter-element coupling and suggests that the array effectively thermalizes during Fe$^+$ ion dose accumulation with transient diffusion processes allowing the magnetic moments to relax into low-energy configurations before freezing. Such extended, low-defect Type-I domain formation is typically observed only when inter-island interactions are sufficiently strong to overcome local disorder and thermal/field-history effects. This spontaneous ordering mechanism yields a degree of magnetic coherence rarely achieved without post-growth treatment in conventionally patterned systems^8,9,27,28^.

Statistical analysis of the vertex configurations extracted from the PEEM images reveals an overwhelmingly dominant population of Type-I vertices (Fig. 3c), confirming that the system naturally adopts its ground-state tiling. The prevalence of low-energy configurations in the *as-prepared* arrays, exceeds that of conventionally fabricated artificial spin ices, reflecting the enhanced uniformity and coupling strength achieved through the embedded architecture. The effective thermalization achieved during ion implantation yields stronger magnetic ordering than typically observed in conventionally fabricated arrays made from $\delta$-doped Pd(Fe)^9,29^ or permalloy^6^, and it is comparable to the behaviour reported in FePd$_{3}$-based ASIs^30^ after post-fabrication treatment.

###  Resonant X-ray scattering

Diffraction is an alternative probe to microscopy for studying the arrays, yielding direct information on correlations. We start by considering nonresonant scattering using an X-ray energy of 690 eV. At this energy, the measurement is sensitive only to charge (electron density) correlations. The difference between the incident ${\bf k}_i$ and scattered ${\bf k}_f$ wave vectors, ${\bf Q} = {\bf k}_f - {\bf k}_i$, defines the momentum transfer. The scattering vector, ${\bf Q}$, can be decomposed into the orthogonal laboratory-frame components $Q_x$, $Q_y$, and $Q_z$ defined using the diffractometer axes. The $Q_x$ and $Q_y$ components correspond to the in-plane directions (with $Q_y$ aligned with the beam direction), whereas $Q_z$ represents the out-of-plane direction with respect to the sample surface. The specific dimensions of the mesospin islands directly determine the scattering intensity distribution in reciprocal space. The well-defined lateral periodicities of the array, give rise to sharp peaks in $Q_x$ and $Q_y$ but since the vertical extent of the islands is finite and not periodic, the scattered intensity manifests as rods of scattering as a function of $Q_z$ with an intensity profile resembling that of the reflectivity shown in Fig. 2.

**Fig. 5:**
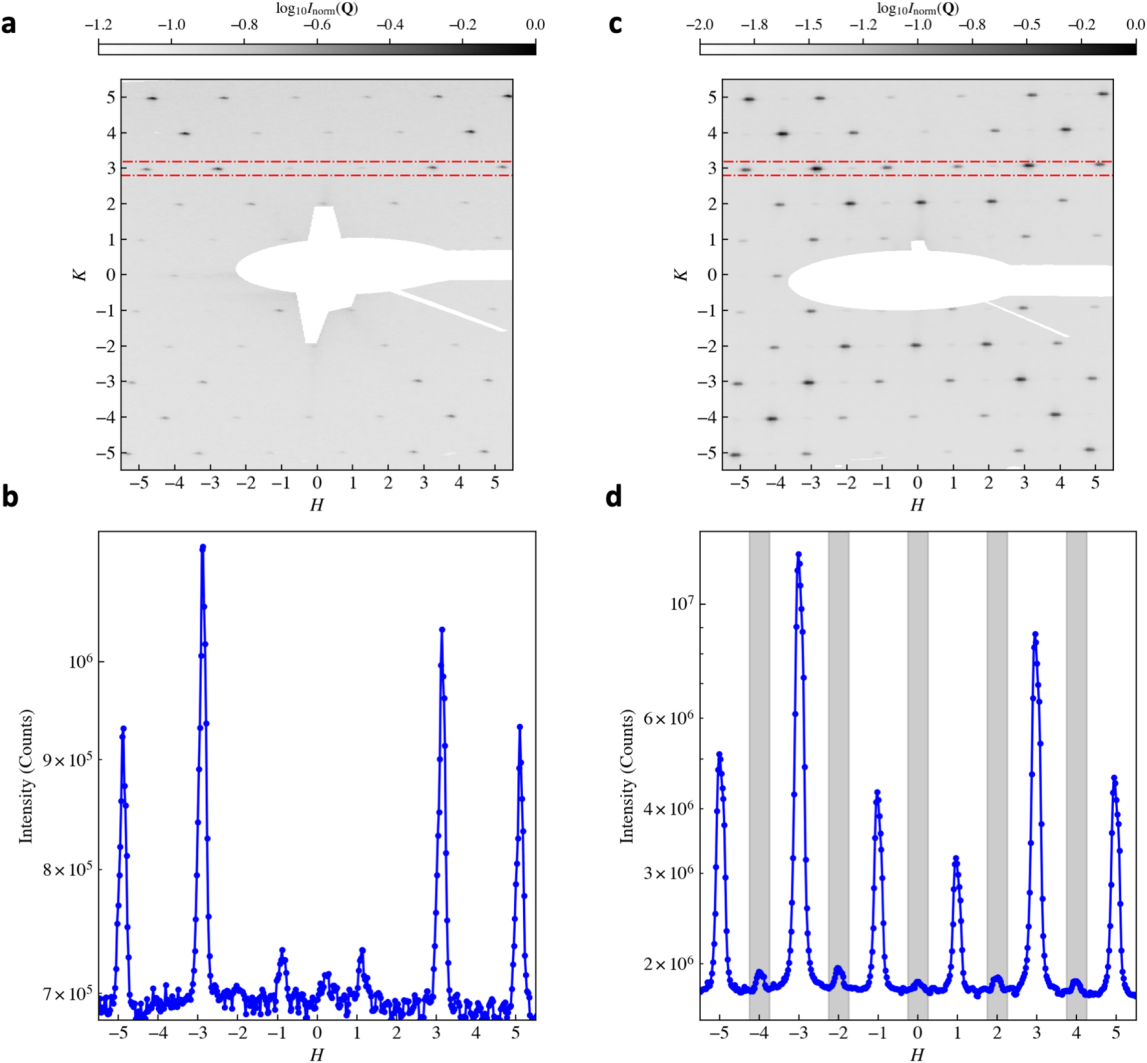
Off- and on-resonance scattering. (**a**) Diffraction pattern of the implanted ASI lattice, shown after corrections for the Ewald sphere curvature. Only charge scattering contributes at this energy, resulting in weak peak intensities and a narrow dynamic range due to the low electron density contrast between Fe-implanted regions and the Pd matrix. The characteristic ”$\times$”-shaped envelope reflects the structural form factor of the mesospin basis. The white shaded area corresponds to the beam stop covering the specular reflection. (**b**) The integrated intensity along *K* as a function of *H* for a region of interest centred on $K=3$ and marked with the red dash-dotted line in (**a**). (**c**) Resonant enhancement increases the structural Bragg peak intensity and reveals additional reflections associated with long-range antiferromagnetic order. The clearer ”$\times$”-shaped modulation highlights the coherent mesospin basis form factor, while the emergence of mixed-parity peaks evidences the magnetic contribution to the scattering. (**d**) The integrated intensity as a function of *H* for a region of interest centred on $K=3$ and marked with the red dash-dotted line in (**c**). Gray-shaded areas denote the positions of the reflections arising due to the antiferromagnetic order on the ASI lattice (Fig. 3a).

A geometric interpretation of the diffraction condition is provided by the Ewald construction (see Fig. 4). In our experiment, diffraction patterns were recorded as two-dimensional CCD images at fixed incidence angle ($\theta ={15}^{\circ }$), which sample a curved reciprocal-space slice determined by the Ewald sphere and detector plane. We did not perform coupled $\theta$–$2\theta$ rocking scans to integrate the full truncation-rod intensity. As a result, diffracted intensity is recorded at discrete pixel locations on the detector (Supplementary Figure). By combining the diffractometer geometry—including the sample-to-detector distance and angular coordinates—with the pixel positions, the detector coordinates were converted into the laboratory-frame momentum transfer vectors ${\bf Q}$. With additional knowledge of the sample periodicity, a further transformation was then applied to express the scattering data in reciprocal lattice units, enabling direct identification of the corresponding (*H*, *K*) indices of the array’s reciprocal lattice (see Supplementary Information). After this conversion, any shifts of the recorded peak positions away from integer values of *H* or *K* can be attributed to uncertainties in the diffractometer angles, sample-to-detector distance, or sample orientation, all of which influence the final transformation to reciprocal lattice units^31^.

A representative diffraction pattern from the implanted array is shown in Fig. 5a. A prominent feature is the abundance of diffraction peaks, which occur at positions where both *H* and *K* are either simultaneously even or odd. These intensity peaks reflect the structural in-plane periodicity. Although at this energy the scattering contrast between the Pd matrix and the Fe-implanted regions is relatively low, resulting in only modest peak intensities, the diffraction peaks remain sharp, indicating long-range structural coherence across the array. The diffraction pattern intensities exhibit mirror symmetry with respect to the *H* axis but not the *K* axis. This asymmetry in *K* originates from the geometric coupling between the out-of-plane momentum transfer $Q_z$ and the in-plane component $Q_y$, which arises from the curvature of the Ewald sphere projection onto the flat detector. The corresponding line scan, integrated region of interest centered on $K = 3$ (red dashed-dot rectangles in Fig. 5a), further confirms the modest peak intensities, their sharpness, and the absence of any half-order reflections. Immediately apparent in the line scan is the mirror symmetry with respect to *H*, as well as a pronounced and non-trivial dependence of the peak intensities with the diffraction order.

**Fig. 6:**
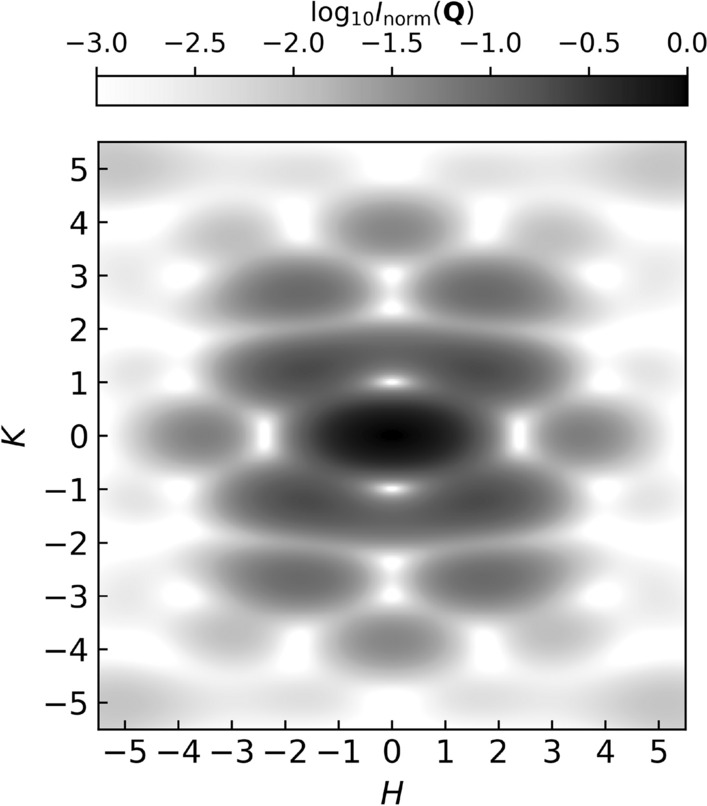
Mesospin form factor. Calculated structural form factor $|F({\bf Q})|$ for the four-mesospin basis forming a square-ASI vertex, using stadium-shaped mesospins as defined in the sample details ($L={470}$ nm, $W={170}$ nm). The reciprocal-space axes are expressed in ASI lattice units.

A striking feature observed in Fig. 5a is the characteristic “$\times$”-shaped distribution of scattered intensity centred at (0, 0). This pattern can be understood within the framework of the convolution theorem, by which the diffracted amplitudes from a crystal are given by the Fourier transform of the real-space lattice multiplied by the Fourier transform of the basis. The measured intensity is then the amplitude squared. In the present case, the basis consists of two stadium-shaped mesospins within each unit cell, defined by their specific spatial arrangement and orientation^32^. To model this contribution, each mesospin was represented by a stadium geometry consistent with the sample details: a rectangle of length $L-W$ and width *W*, capped by semicircles of diameter *W*, with $L={470}$ nm and $W={170}$ nm. The square of the calculated Fourier transform of this basis, expressed in reciprocal lattice units, is shown in Fig. 6. The intensity distribution exhibits enhanced scattering along the diagonal directions, consistent with the experimentally observed patterns. This demonstrates how the morphology and arrangement of the array elements modulate the scattering envelope and imprint a directional anisotropy of the intensity in reciprocal space. This effect is further amplified at resonance due to the increased scattering contrast and the concomitant enhanced visibility of the basis itself. Notably, such a clear manifestation of the basis form factor has not been reported in lithographically patterned square ASI lattices^20,33,34^, where fabrication-induced variations in element shape and size may obscure interference effects associated with the basis geometry^35^. In contrast, ion implantation, as reported here, produces more morphologically uniform mesospins, yielding a better and well-defined scattering envelope. We note that Fig. 6 is intended as a basis-form-factor calculation in the present indexing convention, using stadium-shaped mesospins corresponding to the implanted geometry; its purpose is to illustrate the origin of the reciprocal-space envelope, rather than to provide a full detector-level simulation.

Tuning the X-ray energy to the Fe $L_3$ absorption edge significantly alters the measured diffraction pattern, as shown in Fig. 5c. Together with the Fe $L_{2,3}$ absorption spectrum (Supplementary Figure), this on/off-resonance contrast directly confirms Fe sensitivity: at resonance the total counts increase and magnetic peaks emerge, whereas off resonance they are absent. The resulting magnetic Bragg peaks, which arise from the long-range antiferromagnetic order of the islands, appear at reciprocal lattice positions where *H* and *K* are mixed odd and even (see Supplementary Information). A closer inspection of the line scans in Fig. 5 reveals the markedly different evolution of charge and magnetic peak intensities when moving on and off resonance. Off resonance (Fig. 5b), only the structural reflections are present and their relative intensities follow the envelope imposed by the mesospin form factor, with no features at mixed-parity positions. Upon tuning to the Fe $L_3$ edge (Fig. 5d), the structural peaks increase moderately due to the enhanced contrast between Fe-rich and Fe-poor regions, whereas the mixed-parity reflections appear exclusively at resonance and with intensities that scale with the magnetic contribution to the scattering factor. These magnetic peaks remain weaker than neighbouring structural peaks, consistent with the smaller magnetic scattering cross section and with the geometry of the experiment, which selects only the magnetization components parallel to $Q_y$. The systematic absence of these reflections off resonance, combined with their appearance at the symmetry-expected positions and their intensity scaling, unambiguously identifies them as magnetic Bragg peaks rather than artefacts of higher harmonics or multiple scattering. The relative charge–to–magnetic peak ratios are also in qualitative agreement with the kinematic simulations discussed below, which include both the mesospin form factor and the probe sensitivity axis, supporting their assignment to the Type-I antiferromagnetic ground state.

**Fig. 7:**
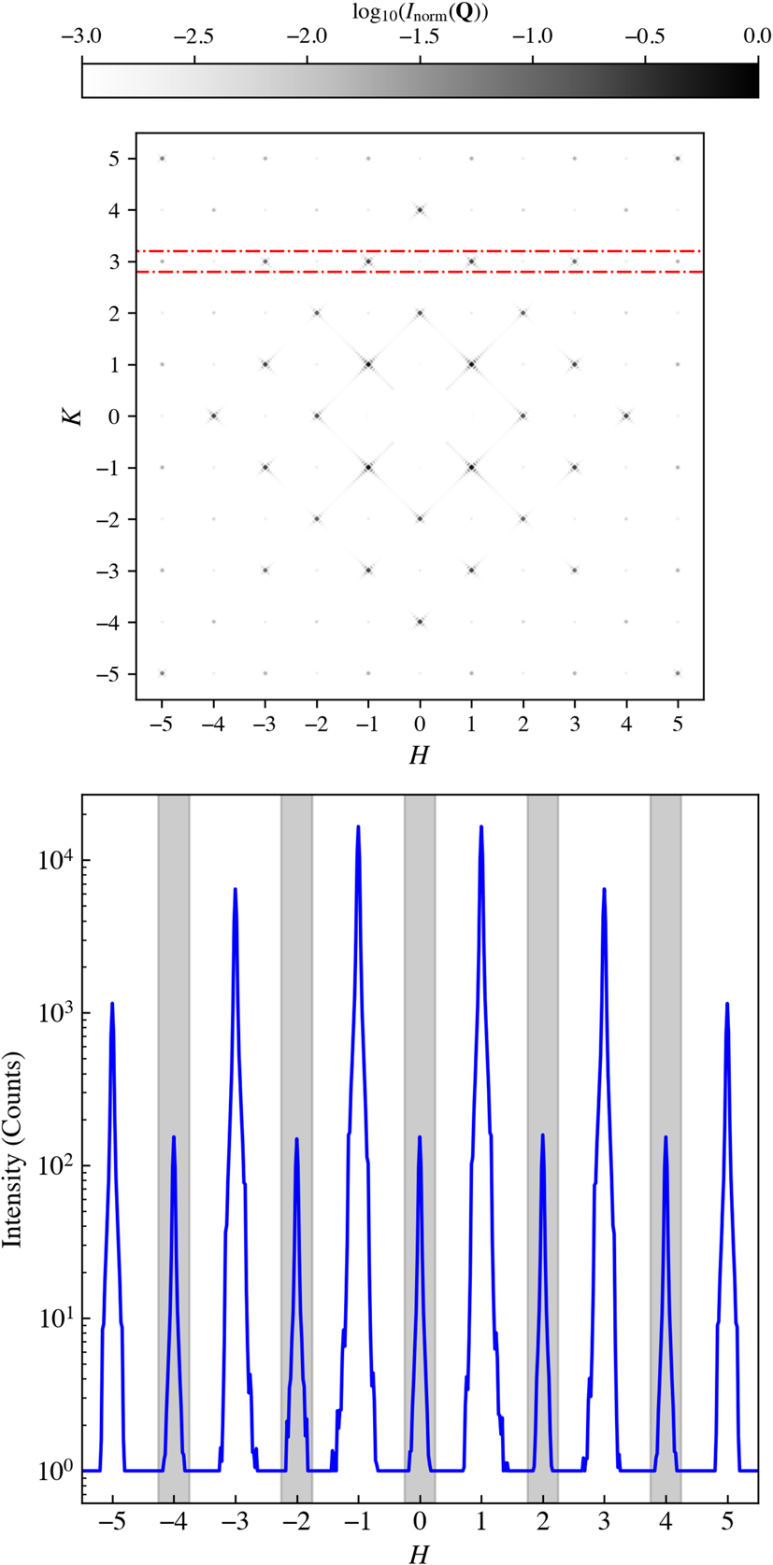
Simulated line scans for the Type-I antiferromagnetic ground state. Calculated intensity profiles, comparable to Fig. 5b,d (along *H* around $K\approx 3$), include structural and magnetic contributions together with the mesospin form factor and X-ray sensitivity axis, and capture the overall experimental intensity pattern. A signal floor at $10^{0}$ was included to better compare against the experiment, where a noise floor is present.

Finally, for completeness, Fig. 7 presents simulated line scans from a Type-I tiling of the islands, extracted in a geometry comparable to Fig. 5b,d. The model combines the calculated structural and magnetic contributions, the mesospin form factor, and the sensitivity axis of the incident X-rays. The contribution from the form factor is accounted for as:

1 $$\begin{aligned} I({\bf Q}) = |A({\bf Q})|^2 = |S({\bf Q})|^2 \, |F({\bf Q})|^2 , \end{aligned}$$

Here $S({\bf Q})=\sum _{j=1}^{N} s_j e^{i{\bf Q}\cdot {\bf r}_j}$ is the complex lattice-sum (structure-factor amplitude), and $|S({\bf Q})|^2$ is the corresponding intensity structure factor, while $F({\bf Q})$ is the mesospin form factor^36^. It is worth noting that, in the most general case, one should account for two distinct form factors: a structural form factor, reflecting the Fe implantation profiles, and a magnetic form factor, describing the magnetization texture within each mesospin. The resulting line scans of Fig. 7 reproduce the overall intensity modulation observed experimentally. We note that the higher-order reflection intensities are sensitive to the effective mesospin dimensions entering the basis form factor, since in implantation-defined mesospins the relevant scattering volume is set by the implanted concentration and magnetization profile rather than by an ideal sharp lithographic boundary^26^. Accordingly, modest changes in the effective mesospin extent can redistribute intensity at larger $|{\bf Q}|$, including the visibility of reflections around $H=\pm 5$ in line cuts such as those shown in Fig. 7. We therefore regard the present comparison as qualitative: the model captures the dominant symmetry, the basis-form-factor envelope, and the probe-sensitivity selection effects, while a detailed one-to-one comparison would require additional modelling beyond this kinematic treatment, including the Ewald-sphere projection onto the planar detector, the intensity profile along the truncation rods sampled by the fixed-angle geometry, and instrumental broadening / resolution effects.

Both experimental and simulated diffraction patterns reveal a pronounced ${\bf Q}$ modulation of the scattered intensity. This modulation reflects the mesospin form factor and the sensitivity of resonant scattering to specific magnetization components. This offers a direct link between local geometry and global diffraction symmetry. The island shape and sensitivity axis of the technique lead to systematic absences and a non-trivial intensity dependence on diffraction order. Careful selection of ${\bf Q}$ values of interest is, therefore, crucial for identifying the most appropriate experimental geometry. Such an approach allows for targeted data acquisition that captures length-scale information directly associated with specific ${\bf Q}$ values and access information that is often underutilized in ASI studies. Historically, analyses of scattered intensities in ASIs have tended to be qualitative or restricted to a limited set of reflections accessible in default measurement geometries, often overlooking features that encode key information about magnetic correlations at other relevant length scales^33,37–39^. Expanding access to a broader region of reciprocal space, combined with kinematic scattering simulations (structure-factor modelling) and a clear link to the reciprocal lattice of the sample itself will enable future experiments to fully exploit the temporal and spatial coherence of resonant soft X-ray scattering. This advancement, in turn, promises unprecedented insight into collective ordering and dynamical processes in ASIs and magnetic metamaterials more generally. It also allows going beyond static snapshots, toward a deeper and more quantitative understanding, enabled by the more consistent and uniform mesospins produced by ion implantation.

##  Conclusions and outlook

Our combined use of resonant X-ray and neutron scattering, together with magnetic microscopy, reveals that ion implantation enables the formation of magnetically ordered metamaterials with structural and magnetic coherence extending over large areas. The implanted Fe ions generate a confined ferromagnetic profile within the Pd host by inducing polarization in the surrounding matrix. This localized magnetic region forms the building blocks of a class of *embedded mesospins*, whose coupling and geometry drive the spontaneous emergence of collective order.

Magnetic imaging confirms that each mesospin acts as a single-domain element and that the implanted islands organize into extended antiferromagnetic domains corresponding to the Type-I ground state of the square geometry. This long-range order emerges directly during the implantation process, without the need for post-fabrication annealing or magnetic field cycling. Our observations suggest that the local energy input, Fe$^+$ ion dose accumulation, and diffusion dynamics during implantation effectively promote self-thermalization, allowing the system to explore low-energy configurations as magnetic moments build up within the mesospins.

In reciprocal space, sharp structural and magnetic Bragg peaks testify to the high morphological uniformity of the implanted array. The scattering envelope obtained demonstrates that implantation overcomes the disorder and variability inherent to lithographic patterning, enabling access to quantitatively interpretable structure–factor regimes that might have been previously inaccessible in artificial spin systems. Comprehensive modeling provides valuable insight that will be instrumental in future sample design and experimental strategy, enabling optimized use of available scattering geometries and detector configurations.

These results establish ion implantation as a scalable, additive approach to realize self-organized magnetic order in structurally coherent metamaterials. The coupling between magnetic and morphological coherence provides a pathway toward functional platforms for spin-resolved and photon-mediated phenomena, including spin–photon coupling, orbital-angular-momentum scattering, and magneto-optical interference in structured media^19,20^. More broadly, the spontaneous emergence of the ground state in an as-fabricated system highlights a potentially useful paradigm in artificial magnetism: magnetic metamaterials that evolve toward their ordered configurations during synthesis. Furthermore, ion implantation offers a unique avenue for directly engineering both structural and functional properties. Through careful selection of ion species, implantation energy and fluence, it becomes possible to tailor local magnetic anisotropy, ordering temperature, and moment magnitude^24–26^—parameters that are typically less independently tunable in lithographically defined systems. Relative to lithographic ASIs, where the main practical tuning parameters are island geometry, thickness, spacing, and deposited material choice, implantation-defined ASIs provide an additional and partly independent set of materials-design knobs through ion species, implantation energy, and fluence. These parameters determine not only the lateral patterning through the mask, but also the local alloy composition and depth profile of the mesospins, enabling control over moment density, ordering temperature, and potentially anisotropy through local chemistry and strain. A practical comparison of these tuning dimensions is summarized in Table 1. Intriguingly, one could envisage extending this concept toward multi-ion or spatially graded 3D architectures, where controlled implantation profiles generate programmable magnetic functionalities or coupled responses across multiple length scales and dimensions. This capability establishes ion implantation not merely as a fabrication route but as a materials design tool for spatially resolved control of emergent magnetic and electronic behavior. Such systems have the potential to bridge the gap between materials design and dynamic self-organization, offering different opportunities for reconfigurable logic, magnonic information processing^14,40,41^, and optical or scattering-based read-out schemes^42,43^ in unconventional computing architectures^44,45^.

**Table 1:** Practical tuning knobs in lithographic and implantation-defined ASIs.

Aspect	Lithographic ASI	Implantation-defined ASI
Mesospin definition	Topographically patterned islands	Embedded magnetic volumes in continuous host
Main geometric knobs	Length, width, thickness, spacing, lattice symmetry	Mask geometry, spacing, lattice symmetry
Main materials knobs	Deposited material / multilayer stack	Ion species, implantation energy, fluence
Most directly tuned quantities	Shape anisotropy, dipolar coupling, volume	Local composition, depth profile, moment density, ordering temperature
Dominant disorder source	Edge roughness, thickness variation, lift-off variability	Implantation straggle / profile gradients; reduced magnetic-edge disorder
Distinctive capability	Direct shape engineering	Spatially graded / multi-species functional patterning in planar host

## Methods

###  Sample fabrication

Initially, a continuous Pd film was deposited by DC magnetron sputtering onto a MgO substrate, with a 5 nm V adhesion layer and a 6 nm Cr capping layer that is in place to avoid ablation of the underlying Pd during ion implanting. Subsequently, a square ASI lattice was fabricated by implanting 30 keV $^{56}$Fe$^{+}$ ions with a nominal fluence of $4 \times 10^{16}$ ions/cm$^{2}$ into a 60 nm Pd film through a patterned Cr implantation mask that was subsequently removed using a wet etch. The Cr mask was defined lithographically prior to implantation; however, the magnetic elements themselves are not topographically patterned. This additive fabrication process results in Fe$_{x}$Pd$_{100-x}$ (where *x* is in atomic percent) ferromagnetic structures embedded within the otherwise non-magnetic Pd matrix. The implanted Fe exhibits both lateral and vertical concentration profiles^26^, the extent of which is controlled through the patterned Cr mask and the implantation energy. After mask removal, the sample surface remains nearly planar; this suppresses the edge-roughness and thickness-variation disorder typical of free-standing lithographic islands while retaining lithographic control of lateral placement. A detailed description of the fabrication process and material properties is provided by Vantaraki *et al.*^25,26^. The particular ASI array fabricated for this study consists of stadium-shaped elements with dimensions of 470 nm in length and 170 nm in width, with an edge-to-edge gap of $g=$170 nm. A representative SEM image of the array is shown in Fig. 1.

In addition to the patterned film, two continuous Pd films implanted homogeneously with Fe ions were fabricated. The Pd layers had a nominal thickness of either 40 nm or 60 nm and were deposited by DC magnetron sputtering onto MgO substrates, each with a 5 nm V adhesion layer and a 6 nm Cr capping layer. Growth and processing conditions were identical to the films used to create the arrays. The 40 nm film was deposited on a $10\times$ 10 $mm^{2}$ substrate, whilst the 60 nm film was deposited on a $20 \times$ 20 $mm^{2}$ substrate to facilitate polarized neutron reflectometry measurements. Ion implantation of the continuous films was carried out under the same conditions as for the patterned array, using 30 ke V $^{56}$Fe$^{+}$ ions at a nominal fluence of $4 \times 10^{16}$ ions/cm$^{2}$. Owing to their extended geometry, the implanted Fe concentration in these films exhibits only a depth-dependent profile.^24^.

###  Magnetization depth profiling

The element-specific Fe magnetization of the implanted samples was determined using resonant synchrotron reflectivity measurements at the SEXTANTS beamline at the SOLEIL synchrotron^46^. The experiments were performed in a reflection geometry on the 40 nm continuous Fe$^{+}$-implanted Pd film at room temperature, with the magnetization saturated along the X-ray sensitivity axis with an applied magnetic field of 50 mT. The scattered intensity was recorded for left ($I^{L}$) and right ($I^{R}$) circularly polarized light, with the photon energy tuned to the Fe $L_{3}$ edge (707 eV). The resonance energy selection is supported by the Fe $L_{2,3}$ X-ray absorption spectrum shown in Supplementary Information. The sum of the two reflectivity curves, $I^{L} + I^{R}$, and the corresponding asymmetry ratio, $AR = (I^{L} - I^{R})/(I^{L} + I^{R})$, were simultaneously fitted using the GenX software package (version 3.8.2, https://aglavic.github.io/genx/) to extract both the chemical and magnetic depth profiles through their respective scattering length densities^47,48^. Complementary magnetization profiling studies were performed using Polarized Neutron Reflectometry (PNR) with one-dimensional spin analysis at the D17 beamline in ILL, Grenoble. Details of the latter are presented in depth in the Supplementary Information.

### Resonant X-ray scattering and microscopy

The magnetic configuration of the implanted square ASI lattice was determined in real space using photoemission electron microscopy combined with X-ray magnetic circular dichroism (PEEM-XMCD), with measurements carried out at the CIRCE (BL24) beamline of the ALBA synchrotron^49^. The PEEM images were collected at room temperature and in the absence of any external magnetic field. The photon energy was tuned to the Fe $L_{3}$ edge to provide element-specific magnetic contrast. The stadium-shaped elements were oriented at ${45}^{\circ }$ with respect to the incident X-ray beam, allowing unambiguous determination of the magnetization direction for all elements in the lattice within the field of view. The magnetic state was imaged in the as-implanted, virgin configuration.

The lateral magnetic ordering of the implanted ASI lattice was further examined in reciprocal space using soft X-ray scattering experiments conducted at the SEXTANTS beamline of the SOLEIL synchrotron^46^. The measurements were carried out in reflection geometry, with diffraction patterns from the periodic lattice captured using a Charge Coupled Device (CCD) detector. To shield the detector from the intense specularly reflected beam, a beam stop was deployed. A circularly polarized X-ray beam, tuned to the Fe L$_{3}$ edge, provided element-specific sensitivity to the magnetization of the mesospins. The angle of incidence, $\theta$, was set to ${15}^{\circ }$ to enhance sensitivity to the in-plane magnetization. Consistent with the microscopy measurements, the ASI lattice was aligned so that the X-ray beam propagated along the [1, 1] direction of the square array.

### Kinematic scattering simulations

We performed kinematic scattering simulations of the square ASI by constructing a finite Type-I antiferromagnetic lattice in the experimental geometry, with the incident beam aligned along the [1, 1] direction of the array. The charge channel was calculated from the coherent sum of all islands, weighted by a structural basis form factor $F({\bf Q})$ for the two orthogonal stadium-shaped mesospins in each unit cell. Unless otherwise stated, the nominal sample dimensions $L={470}$ nm and $W={170}$ nm were used. Because the implantation-defined mesospins correspond to effective magnetic / structural volumes rather than perfectly sharp topographic boundaries, the highest-order intensities are sensitive to modest variations in the effective mesospin dimensions. The magnetic channel was computed separately from the ordered mesospin moments using the probe sensitivity axis appropriate to the resonant soft X-ray geometry, and the total simulated intensity was obtained by combining the structural and magnetic contributions. Reciprocal-space maps and line cuts were then extracted in the same (*H*, *K*) indexing used for the experimental analysis, with optional masking of the central beam-stop region for direct comparison with the measured diffraction patterns. The code used for these simulations is available at: https://github.com/kapaklis/XRMS_on_sqASIs.git.

## Supplementary Information

Supplementary Information.

## Data Availability

The data that support the findings of this study are available from the corresponding authors upon reasonable request. Neutron scattering data are available at: KAPAKLIS VASSILIOS, CUBITT Robert, SAERBECK Thomas, VANTARAKI Christina, & WOLFF Maximilian. (2024). Magnetic metamaterials produced by ion-implantation (ReMade - TNA) [Dataset]. Institut Laue-Langevin (ILL). DOI:10.5291/ILL-DATA.DIR-329.
