# Supplementary material for: Long-range structural and magnetic coherence in embedded mesospin metamaterials
Source: Sci Rep. 2026 Apr 12;16:12178. doi: 10.1038/s41598-026-48207-w (PMC13076862; doi:10.1038/s41598-026-48207-w)
Supplement: Supplementary file 1 — Supplementary Information. [file 41598_2026_48207_MOESM1_ESM.pdf]

Supplementary Information:

Long-Range Structural and Magnetic Coherence in Embedded Mesospin Metamaterials

X-ray scattering detector images

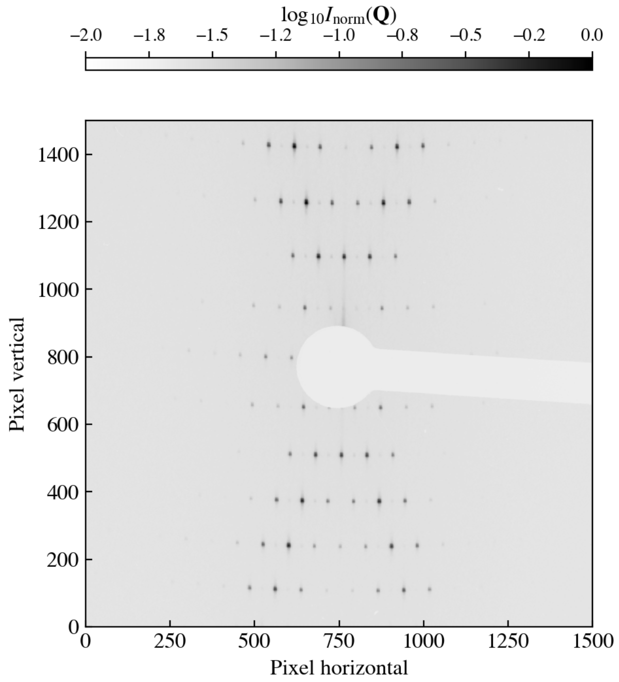

Supplementary Fig. S1 | Raw CCD detector image acquired on-resonance. Unprocessed pixel data from a resonant soft X-ray scattering snapshot. The apparent curvature of the diffraction features arises because the 2D detector captures a curved slice through reciprocal space corresponding to the portion of the Ewald sphere intersecting the Bragg rods.

The raw CCD detector images (Supplementary Fig. S1) already reveal the key features expected for scattering from a square-lattice array under the given experimental geometry. Because the mesospin pitch is on the order of several hundred nanometres, the corresponding diffraction peaks appear close to the specular reflection, which in our case is masked by a beam stop. As the detector samples a curved section of reciprocal space—specifically, the set of reciprocal-space points lying on the portion of the Ewald sphere intercepted or projected onto the detector[S1] (Fig. 4)—the raw images can appear distorted, with peaks following curved trajectories rather than the straight lines expected from a square lattice.

A precise mapping of the pixel coordinates of the image to the corresponding (H,K) values in reciprocal space is essential for quantitative analysis. This mapping can be performed once the exact experimental geometry and beam parameters are known. Importantly, this mapping also compensates

for the distortions introduced by the experimental geometry

Polarized neutron reflectometry

Polarized Neutron Reflectometry (PNR) with one-dimensional spin analysis was performed at the D17 beamline at ILL, Grenoble[S2,S3] on a $20\times 20\,\mathrm{mm}^2$ film of 60 nm Pd implanted with 30 keV Fe^+, under a saturating magnetic field of 100 mT. Measuring the reflectivity of neutron spins parallel (R^+) and anti-parallel (R^-) to the magnetization of the sample allows to extract the magnetic depth profile. The R^+ and R^- reflectivity curves, along with the spin asymmetry $SA=\frac{R^+-R^-}{R^++R^-}$, were fitted simultaneously using the GenX program[S4,S5].

Unlike the X-ray data, the magnetic depth profile obtained from PNR measures the distribution of magnetization in depth and includes contributions from both Fe and Pd. The PNR curves are shown in Supplementary Fig. S2 (b), separately recorded for the two spin channels, R^+ and R^-. For implanted films, where ion mixing produces interdiffused chemical and magnetic gradients, the fit output is most robustly represented by the SLD depth profiles; a decomposition into unique layer thickness and interfacial roughness values is not physically unique. The Spin Asymmetry (SA) is directly proportional to the magnitude of the magnetic moment in the plane of the sample and along the quantisation axis. When fitting, we account for some Cr being implanted into the film as a result of knock-on collisions from the capping layer, based on previous results[S6].

The neutron reflectivity with spins parallel (R^+) and antiparallel (R^-) is fitted to a structural and magnetic model of neutron scattering length densities as a function of depth using the GenX program (version 3.8.2, `https://aglavic.github.io/genx/`). The magnetic SLD is proportional to the in-plane magnetization as a function of depth ($\rho_\mathrm{m} = 2.91044\times 10^{-12}\,\text{Å}^{-2}\mathrm{mA}^{-1}\times M_\perp[\mathrm{Am}^{-1}]$). The spin asymmetry SA, highlighting only the magnetic contribution to the reflectivity, has been fitted simultaneously. Whilst the magnetic profile appears broader, the magnetic moment remains confined to within approximately 15 nm of the surface. Since PNR measures the total magnetic moment, any induced magnetization in the Pd layer extends the magnetic thickness beyond the Fe-implanted region itself.

Lattices and Indexation

A key step in interpreting the experimentally obtained scattering data is to index the diffracted intensities to a suitable lattice that describes the sample itself, rather than relying solely on the reciprocal space

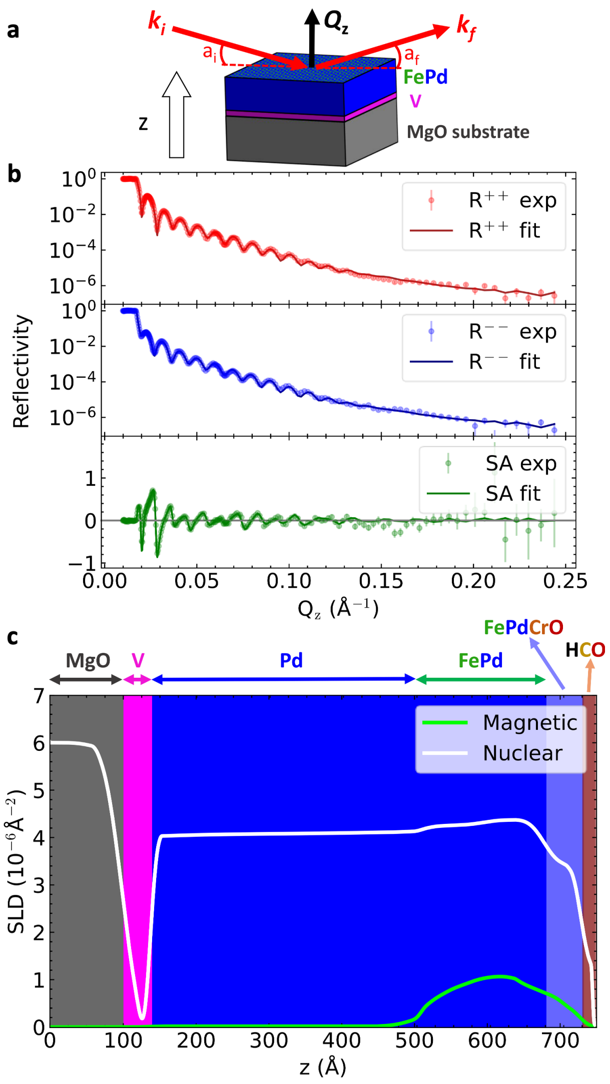

Supplementary Fig. S2 | Polarized neutron reflectivity. a Schematic of the 30 keV Fe^+-implanted film. Polarized neutron reflectometry was performed in specular geometry ($a_i = a_f$), as a function of out-of-plane momentum transfer Q_z. **b** Neutron reflectivity curves for spin-up (R^+) and spin-down (R^-) channels, and the spin asymmetry (SA). Symbols represent experimental data; solid lines correspond to the best-fit model. **c** Derived nuclear and magnetic scattering length density (SLD) profiles. Background shading indicates the MgO substrate, V adhesion layer, Pd film and impurity regions.

defined by the diffractometer axes. The islands themselves are arranged on a simple 2D square Bravais lattice denoted by the vectors $\vec{a}_{1,2}$ (Supplementary Fig. S3). The reciprocal lattice vectors are given by:

$$\vec{a}_1^* = 2\pi \frac{\vec{a}_2 \times \hat{n}}{||A||} \quad ; \quad \vec{a}_2^* = 2\pi \frac{\hat{n} \times \vec{a}_1}{||A||},$$

where $\hat{n}$ is the unit vector normal to the 2D plane and $||A||$ is the magnitude of the scalar triple product $\vec{a}_1 \cdot (\vec{a}_2 \times \hat{n})$. A translation in reciprocal space is given by $\vec{G}_{h,k} = h\vec{a}_1^* + k\vec{a}_2^*$.

When all vertices in the square ASI are of the Type-I configuration, the primitive magnetic lattice is

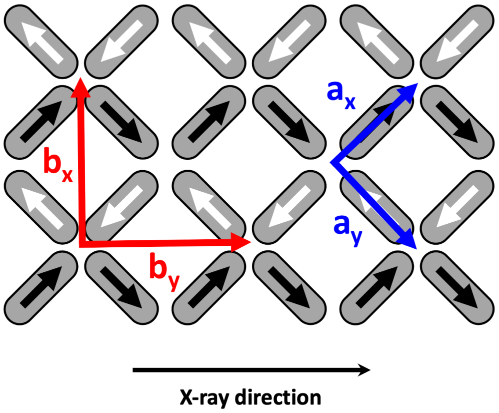

Supplementary Fig. S3 | Real-space lattice vectors. Real-space representation of the square ASI lattice and its magnetic ground state. The mesospin array forms a square Bravais lattice, while the Type-I antiferromagnetic configuration, combined with the X-ray sensitivity axis along the lattice diagonal, results in a magnetic unit cell enlarged by a factor of $\sqrt{2}$ and rotated by 45°, i.e., a $(\sqrt{2},\times,\sqrt{2})$R45° reconstruction. This defines the indexing scheme used to assign structural and magnetic Bragg peaks in reciprocal space.

$\sqrt{2}$ times larger than the Bravais lattice of the islands and is rotated 45° with respect to it, Supplementary Fig. S3. In Wood notation[S7], the magnetic lattice is $\left(\sqrt{2}\times\sqrt{2}\right)R45^\circ$ or in matrix form[S8], $\left[\begin{smallmatrix}1 & \bar{1}\\ 1 & 1\end{smallmatrix}\right]$.

To better visualise reciprocal space, and noting the 45° rotation of the magnetic to island lattice with respect to the islands, we can re-index the lattice using a new set of vectors aligned along the [1,1] direction with basis vectors $\vec{b}_{1,2}$. Here $\vec{b}_1$ is aligned with the [1,1] direction and $\vec{b}_2$ perpendicular to it, along the $[1,\bar{1}]$ direction (Supplementary Figs. S3 and S4). The relationship between the two indexations is simply $\vec{b}_1 = \vec{a}_1 + \vec{a}_2$ and $\vec{b}_2 = \vec{a}_1 - \vec{a}_2$. Normalising these vectors, i.e. $\hat{b}_{1,2} = \frac{1}{\sqrt{2}}\vec{b}_{1,2}$ simplifies the indexing to this new lattice description with the transformation, written in matrix form, as follows:

$$\begin{pmatrix}\vec{b}_1\\ \vec{b}_2\end{pmatrix} = M\begin{pmatrix}\vec{a}_1\\ \vec{a}_2\end{pmatrix} = \frac{1}{\sqrt{2}}\begin{pmatrix}1 & \bar{1}\\ 1 & 1\end{pmatrix}\begin{pmatrix}\vec{a}_1\\ \vec{a}_2\end{pmatrix}, \tag{S1}$$

which in reciprocal space becomes:

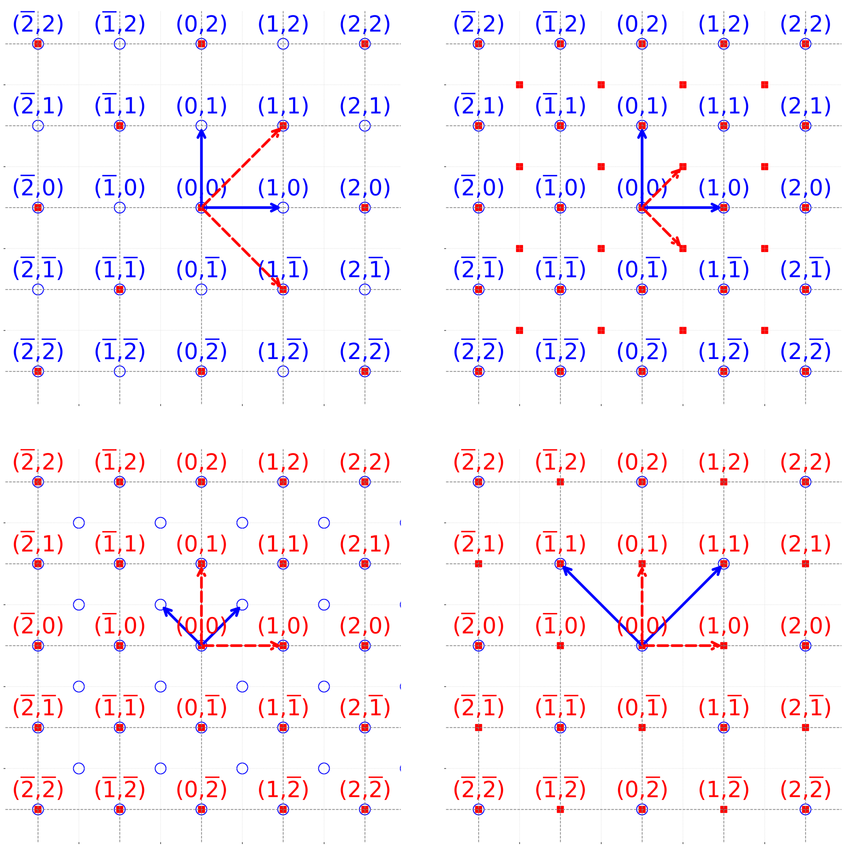

Supplementary Fig. S4 | Reciprocal lattice indexing. Representations of the array lattice in real (left) and reciprocal space (right). The array can be indexed to the simple square Bravais lattice associated with the islands (top) or the $\left(\sqrt{2}\times\sqrt{2}\right)R45^{\circ}$ unit cell of the magnetic Type-I configuration (bottom).

$$\begin{aligned}\begin{pmatrix}\vec{b}_1^* \\ \vec{b}_2^*\end{pmatrix} &= M^* \begin{pmatrix}\vec{a}_1^* \\ \vec{a}_2^*\end{pmatrix} = \left(M^{-1}\right)^{\mathrm{T}} \begin{pmatrix}\vec{a}_1^* \\ \vec{a}_2^*\end{pmatrix} \\ &= \frac{1}{\sqrt{2}}\begin{pmatrix}1 & 1 \\ \bar{1} & 1\end{pmatrix}\begin{pmatrix}\vec{a}_1^* \\ \vec{a}_2^*\end{pmatrix}.\end{aligned} \tag{S2}$$

In this indexing scheme, as shown in Supplementary Fig. S4, structural diffraction from the islands is observed when both H and K are either even or odd. In contrast, magnetic Bragg peaks only appear at positions where H and K are mixed, i.e. one is even and the other is odd.

Under resonant X-ray scattering conditions, however, the magnetic scattering cross section becomes sensitive to the projection of the magnetic moment along the direction of the incident beam. This

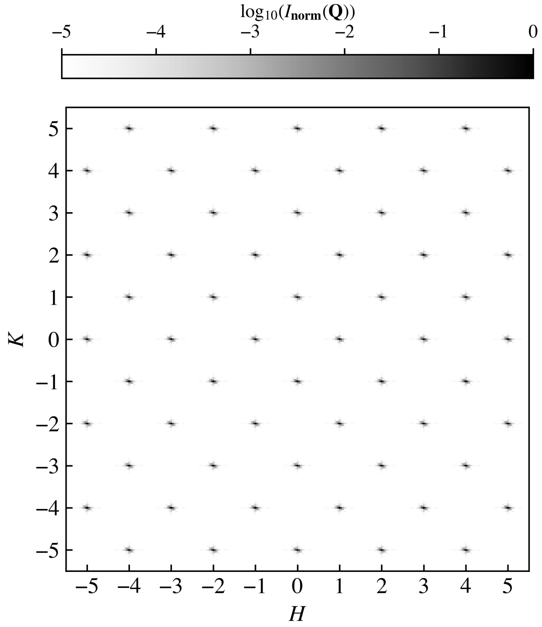

Supplementary Fig. S5 | The average SSF map calculated from the PEEM-XMCD images. Sharp Bragg peaks are seen due to the extended antiferromagnetic order.

directional sensitivity modifies the observed symmetry of the magnetic order when the beam is incident along the [1,1] direction, effectively reducing the symmetry to a 2×1 construction (in terms of $\vec{b}$). Consequently, certain diffraction peaks become systematically absent in this geometry, depending on the parity of H and K.

Spin structure factor maps

The long-range magnetic order observed in the PEEM images (Fig. 3) provides an independent confirmation of the indexing procedure. The square modulus of the Fourier transform of the PEEM data, the spin structure factor (SSF), directly reveals the magnetic lattice in reciprocal space. The Fourier transform of the complete set of microscopy images is shown in Supplementary Fig. S5 and can be indexed to the $\left(\sqrt{2}\times\sqrt{2}\right)R45^{\circ}$ symmetry described above. The presence of sharp diffraction peaks further confirms the long-range magnetic order seen within the field of view of the PEEM instrumentation. Immediately apparent is the lack of any "×" feature (see main text) and no systematic absences. The magnetic configuration of the islands is fully described by the Type-I vertex configuration and its associated symmetry. The binary contrast in the intensities measured in the PEEM data explains the lack of any basis contribution.

Given the experimental evidence for Type-I vertex configurations, we simulate the SSF maps by considering an infinite lattice of mesospin islands with spin S. The scattered intensity is obtained from

the square of the Fourier transform of the spin–spin correlations,

$$I(\mathbf{Q}) = \frac{1}{N^2} \left| \sum_{i,j=1}^{N} \mathbf{S}_i \mathbf{S}_j e^{i\mathbf{Q}\cdot(\mathbf{r}i - \mathbf{r}j)} \right|^2 . \tag{S3}$$

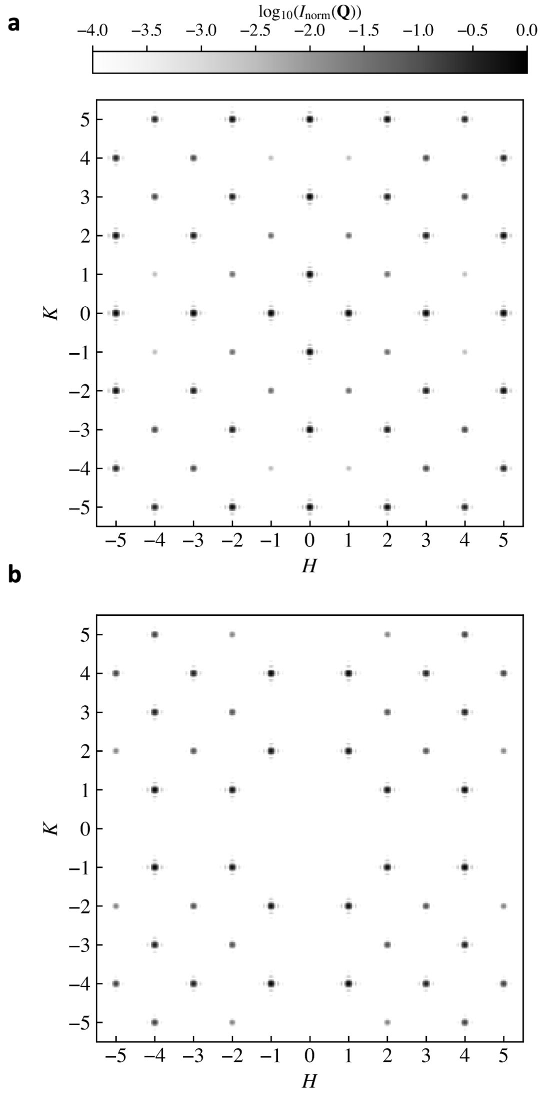

Supplementary Fig. S6 | SSFs for parallel and perpendicular spin components. Simulated SSF maps for an infinite Type-I vertex configuration in a square ASI lattice obtained by considering, **a**: the spin component projected parallel and **b**: the perpendicular to **Q** according to Eq. (S3).

Here N is the number of islands and **r** their real-space position vectors. Historically, neutron scattering has been extensively employed to probe spin-ice systems, with the measured magnetic structure factor determined by the component of the moment *perpendicular* to the scattering vector **Q**, i.e., $S^{\perp}$[S9]. In contrast, resonant X-ray scattering is sensitive to the component of the magnetization *parallel* to **Q**. To assess how this difference in probe sensitivity affects the observed SSF, we calculate Eq. (S3) using either the

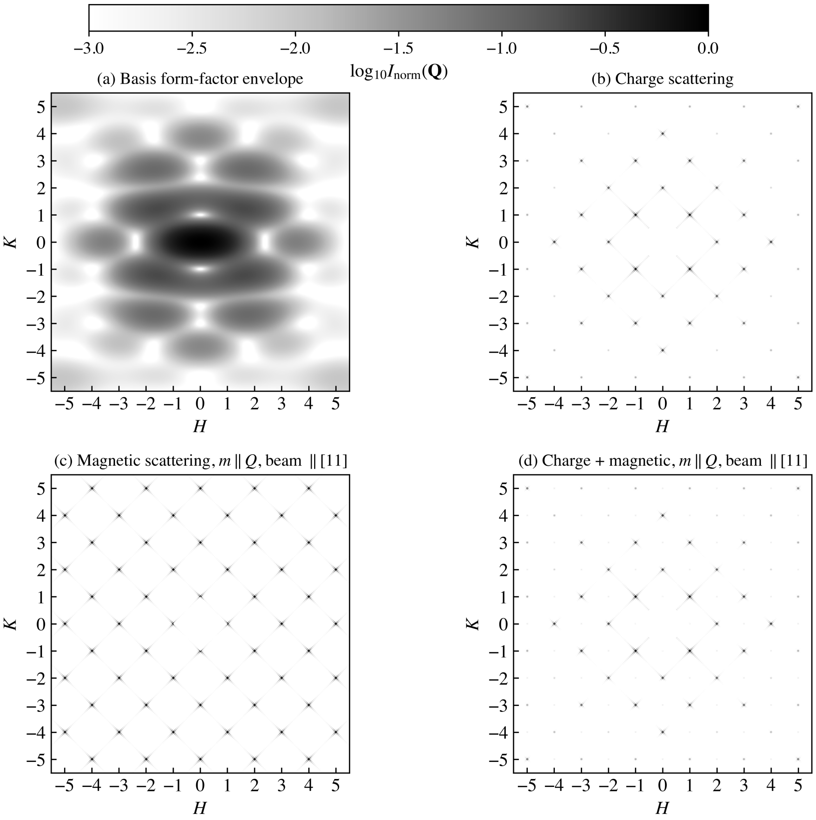

Supplementary Fig. S7 | Simulated scattering patterns for the Type-I state, shown as successive steps of the calculation. (a) Basis form-factor envelope arising from the two-island unit cell and stadium-island geometry. (b) Charge scattering intensity including the island form factor. (c) Magnetic spin structure factor calculated for the spin component projected parallel to the in-plane scattering vector, with Q parallel to [11]. (d) Total simulated resonant scattering intensity obtained from the coherent sum of the charge and magnetic scattering amplitudes.

parallel or the perpendicular spin components and compare the resulting maps in Supplementary Fig. S6.

Systematic absences and/or weak intensities are observed at specific reciprocal lattice points in the simulated SSF maps (Supplementary Fig. S6). In the case where only the spin components parallel to **Q** are considered, the reduced symmetry of the magnetic periodicity changes the selection rules that govern the appearance (or suppression) of the magnetic peaks, as described above. This symmetry dependence highlights the importance of accounting for probe-specific sensitivity when interpreting magnetic scattering data. In particular, understanding these symmetry constraints is essential both for optimizing experimental geometries and for accurately analyzing the intensity distributions in X-ray magnetic scattering patterns.

Simulated scattering patterns

The steps of the scattered-intensity simulation are summarized in Supplementary Fig. S7. The code used for these simulations is available at `https://github.com/kapaklis/XRMS_on_sqASIs.git`.

Fe $L_{2,3}$ x-ray absorption

The x-ray specular reflection of the continuous Fe-implanted sample was measured at the Sextants beamline at the Soleil synchrotron with an incidence angle of 5°. The results are presented in Supplementary Fig. S8. The iron L_3 absorption edge is clearly observed at 706.5 eV, while the L_2 edge appears at 721 eV.

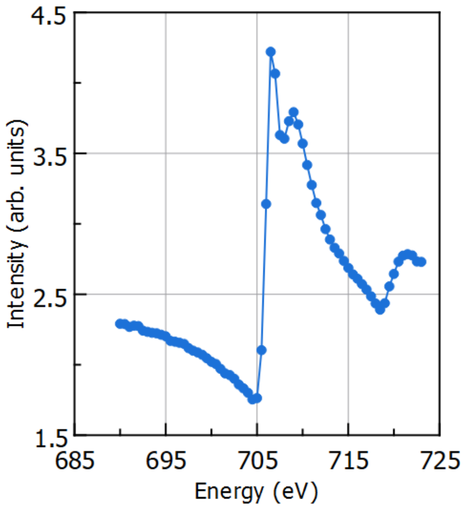

Supplementary Fig. S8 | Intensity of the specular x-ray reflection at an angle of 5° for the Fe-implanted continuous film with nominal 40 nm Pd.

[S1] C. Schlepütz, S. Mariager, S. Pauli, R. Feidenhans'l, and P. Willmott, Angle calculations for a (2+3)-type diffractometer: focus on area detectors, *Journal of Applied Crystallography* **44**, 73–83 (2011).

[S2] T. Saerbeck, R. Cubitt, A. Wildes, G. Manzin, K. H. Andersen, and P. Gutfreund, Recent upgrades of the neutron reflectometer D17 at ILL, *Journal of Applied Crystallography* **51**, 249–256 (2018).

[S3] V. Kapaklis, R. Cubitt, T. Saerbeck, C. Vantaraki, and M. Wolff, Magnetic metamaterials produced by ion-implantation (ReMade - TNA), *Proposal Number DIR-329* (2024), DOI:10.5291/ILL-DATA.DIR-329.

[S4] M. Björck and G. Andersson, GenX: an extensible X-ray reflectivity refinement program utilizing differential evolution, *Journal of Applied Crystallography* **40**, 1174–1178 (2007).

[S5] A. Glavic and M. Björck, GenX 3: the latest generation of an established tool, *Journal of Applied Crystallography* **55**, 1063–1071 (2022).

[S6] C. Vantaraki, K. Ignatova, D. Moldarev, M. P. Grassi, M. Foerster, D. Primetzhofer, U. B. Arnalds, and V. Kapaklis, Magnetic texture control in ion-implanted metamaterials, *Physical Review Materials* **9**, 084402 (2025).

[S7] E. A. Wood, Vocabulary of Surface Crystallography, *Journal of Applied Physics* **35**, 1306 (1964).

[S8] R. L. Park and H. H. Madden, Annealing changes on the (100) surface of palladium and their effect on CO adsorption, *Surface Science* **11**, 188 (1968).

[S9] S. T. Bramwell and M. J. P. Gingras, Spin Ice State in Frustrated Magnetic Pyrochlore Materials, *Science* **294**, 1495 – 1501 (2001).
